# Hydroxypropyl methylcellulose, a viscous soluble fiber, reduces insulin resistance and decreases fatty liver in Zucker Diabetic Fatty rats

**DOI:** 10.1186/1743-7075-9-100

**Published:** 2012-11-12

**Authors:** David A Brockman, Xiaoli Chen, Daniel D Gallaher

**Affiliations:** 1 Department of Food Science and Nutrition, University of Minnesota-Twin Cities, 1334 Eckles Avenue, St. Paul, MN 55108-1038, USA

**Keywords:** Acylcarnitines, Adiposity, Dietary fiber, Fatty liver, Insulin resistance, Viscosity

## Abstract

**Background:**

Diets producing a high glycemic response result in exaggerated insulin secretion which induces hepatic lipogenesis, contributing to development of insulin resistance and fatty liver. Viscous dietary fibers blunt the postprandial rise in blood glucose, however their effect on type 2 diabetes and obesity are not entirely known. This study examined the effect of chronic consumption of the viscous, non-fermentable dietary fiber, hydroxypropyl methylcellulose (HPMC), on glucose control, insulin resistance and liver lipids in an obese diabetic rat model.

**Methods:**

Three groups of Zucker Diabetic Fatty (ZDF) rats were fed diets containing either 5% non-viscous cellulose (control), low viscosity HPMC (LV-HPMC) or high viscosity HPMC (HV- HPMC) for six weeks. Zucker lean littermates consuming cellulose served as a negative control. Markers of glucose control, including oral glucose tolerance test, glycated hemoglobin and urinary glucose, were measured as well as adiposity and the accumulation of liver lipids.

**Results:**

The HPMC diets increased the viscosity of the small intestinal contents and reduced the postprandial rise in blood glucose. The food efficiency ratio was greater with HPMC feeding compared to the obese control and urinary excretion of glucose and ketone bodies was reduced. The two HPMC groups had lower glycated hemoglobin and kidney weights and a reduced area under the curve during a glucose tolerance test, indicating improved glucose control. Epididymal fat pad weight as percent of body weight was reduced in the HV-HPMC group compared to the obese control group. The HV-HPMC group also had lower concentrations of liver lipid and cholesterol and reduced liver weight. However, HV-HPMC feeding did not affect hepatic gene expression of SREBP-1c or FAS. Muscle concentration of acylcarnitines, a lipid intermediate in fatty acid β-oxidation, was not different between the HPMC groups and obese control, suggesting no change in muscle fatty acid oxidation by HPMC.

**Conclusions:**

Consumption of the viscous non-fermentable fiber HPMC decreased diabetic wasting, improved glucose control and reduced insulin resistance and fatty liver in a model of obesity with diabetes.

## Background

Diabetes is a major and growing public health problem in the United States, currently estimated to affect 8.3% of the U.S. population [1], the vast majority of whom have type 2 diabetes. Type 2 diabetes, which is strongly associated with obesity, is characterized by the presence of chronic hyperglycemia as a result of insulin resistance. Other common features include fatigue, weight loss, glycosuria and ketonuria. A common pathogenic event accompanying obesity with insulin resistance is the development of fatty liver, which is the earliest manifestation of nonalcoholic fatty liver disease (NAFLD) [2]. The rapid increase in recent years in the prevalence of NAFLD parallels the increase in obesity, insulin resistance and type 2 diabetes [3].

One strategy to decrease insulin resistance and adiposity, and therefore ameliorate the pathogenic consequences of these conditions, may be to use foods or food components that slow the intestinal absorption of glucose from a meal, resulting in a blunted postprandial glucose response. Consumption of diets that elicit a lower postprandial glucose curve improve insulin sensitivity and reduce total fat mass in both rats [4] and humans [5,6]. Hyperglycemia, resulting from rapidly absorbed glucose from a meal, will greatly stimulate insulin secretion, causing a large increase in plasma insulin. This exaggerated insulin response to a meal will alter the normal homeostatic control of plasma glucose and non-esterified fatty acids and increase glycogenesis and lipogenesis in the liver as well as increase glucose uptake by insulin-sensitive tissues. In contrast, a slower absorption of glucose was found to decrease the insulin response and decrease hepatic lipogenic gene expression [7].

A reduced postprandial glucose response has been demonstrated with many types of viscous dietary fibers, including guar gum [8], psyllium [9] and hydroxypropyl methylcellulose (HPMC) [10]. They appear to do so through delayed gastric emptying, reduced access of digestive enzymes to their substrates, and reduced diffusion to the absorptive surface of the intestine [11,12]. A majority of soluble fibers are also readily fermented, producing short-chain fatty acids (SCFA) that are absorbed by the large bowel. SCFAs can increase the secretion of appetite-related hormones such as peptide YY (PYY) and glucagon-like peptide-1 (GLP-1) from the intestinal tract [13,14], increasing satiety and perhaps decreasing gut motility [14,15]. To further examine the role of dietary fiber-mediated intestinal viscosity on improving glucose control and decreasing insulin resistance, without the potentially confounding effect of fiber fermentation, we utilized a viscous non-fermentable fiber, HPMC. In addition to assessing glucose control and insulin resistance, we also examined adiposity, hepatic lipids and the expression of genes related to metabolic fuel utilization. For these studies we employed an animal model of obesity with type 2 diabetes, the Zucker Diabetic Fatty (ZDF) rat. This diabetic phenotype of the Zucker rat originated from selective breeding of hyperglycemic Zucker rats, which possess a defective leptin receptor, and typically develops into type 2 diabetes by 10 weeks of age. Herein we report that HPMC profoundly improves glucose control, reduces visceral adiposity and decreases the accumulation of liver lipids in this animal model of obesity with type 2 diabetes.

## Methods and materials

### Animals

Male ZDF rats and their lean littermates were purchased at six weeks of age from Charles River Laboratories (Wilmington, MA) and housed individually in an environmentally controlled room (22°C) with a 12 hour light–dark cycle. Rats were given free access to food and water. Animal handling and housing followed National Institutes of Health guidelines and experimental procedures were approved by the University of Minnesota Animal Care and Use Committee.

### Diet composition

Rats were adapted to the control diet, modified from the AIN-93G diet [16], for five days before assignment to the study diets. The composition of the control diet was as follows (g/kg): casein, 200; cornstarch, 449.5; sucrose, 100; soybean oil, 120; cellulose, 80; mineral mix, 35; vitamin mix, 10; L-cysteine, 3; choline bitartrate, 2.5; and tert-butylhydroquinone, 0.014. Cellulose is a non-fermentable and non-viscous dietary fiber. HPMC, a synthetic, non-fermentable fiber available in different viscosity grades, was used to formulate the two viscous fiber diets. In the HPMC groups a portion of cellulose (5% of total diet weight) was replaced with either a low viscosity HPMC (LV-HPMC) or a high viscosity HPMC (HV-HPMC). The low viscosity HPMC was a mixture of 25% K100-LV Methocel and 75% K3-LV Methocel, and the high viscosity HPMC was a mixture of 50% K4M Methocel and 50% K15M Methocel. HPMC was a gift from the Dow Chemical Company (Midland, MI). As the total dietary fiber concentration was 8% for all diets, the percentages (by weight) of digestible carbohydrate, protein and fat remained constant and the diets were therefore isoenergetic. Zucker lean littermates (n=12) were fed the control diet as a negative control and the three ZDF obese groups (n=12/group) were fed either the control diet, LV-HPMC diet or HV-HPMC diet.

Diets were fed for six weeks. After five weeks of feeding, a 24-hour fasting urine sample and a blood sample from the retro-orbital sinus were collected. Blood was centrifuged and plasma collected and stored at −80°C until analyzed. At six weeks of feeding, rats were fasted overnight, and the following morning presented a 5 g meal of their respective diet. Rats consumed >95% of this meal. Two and a half hours after the meal was presented, rats were anesthetized with isoflurane and blood collected by cardiac puncture following laparotomy into syringes containing EDTA (1 mg/mL) and plasma collected after centrifugation. Small intestinal contents were collected from the small intestine by finger stripping. The epididymal, combined retroperitoneal and perirenal (retroperitoneal+perirenal) and inguinal fat pads, liver, kidney and gastrocnemius muscle were excised, weighed, flash frozen in liquid nitrogen and stored at −80°C.

### Intestinal content viscosity

Small intestinal contents were held at 4°C until processing, which was done within six hours of collection. Small intestinal contents viscosity was determined as described by Islam et al. [17]. Viscosity measurements are expressed as millipascals seconds (mPa·s).

### Plasma glucose, non-esterified fatty acids, insulin, corticosterone, and adipokines

Plasma glucose was measured in whole blood using a glucometer (AlphaTrak, Abbott Laboratories, Abbott Park, IL) calibrated for rodents. Plasma non-esterified fatty acids (NEFA) were measured in 24-hour fasted (five weeks) and fed (six weeks) rats using an enzymatic kit (WAKO Diagnostics, Richmond, VA). Fasting plasma insulin and leptin were measured at five weeks using rat-specific radioimmunoassay kits (Millipore, Billerica, MA) and fasting adiponectin measured at five weeks with a rat-specific single-plex kit used on a Luminex system (Millipore). Plasma corticosterone was measured at six weeks by ELISA using a commercial kit after extraction of the plasma, as per the manufacturer’s instruction (Oxford Biomedical Research, Rochester Hills, MI). The quantitative insulin sensitivity check index (QUICKI) was used to estimate insulin sensitivity [18], where QUICKI = 1/[log(fasting insulin (μU/mL))+log(fasting glucose (mg/dL))]. The percentage of glycated hemoglobin was determined at five weeks using an affinity column (Glyco-Tek, Helena Laboratories, Beaumont, TX).

### Meal and glucose tolerance tests

Meal tolerance tests were performed after one week on the diet. Rats fasted for 12 hours had their blood glucose measured and then were presented with 5 g of their respective diet for 20 min, after which the remaining diet was removed. The amount of the meal consumed did not differ among the groups (data not shown). Blood glucose was measured at 15, 30, 60, 90 and 120 min after the meal was first presented. In order to capture differences in fasting blood glucose concentrations among the groups, total area under the curve (tAUC) was calculated, using a blood glucose concentration of zero as a baseline, by the trapezoidal rule. Oral glucose tolerance tests (OGTT) were performed on 12-hour fasted rats after five weeks on the diet using 0.5 g/kg glucose administered by gavage. Plasma glucose was measured at 0, 15, 30, 60, 90 and 120 min and both the tAUC and the incremental area under the curve (iAUC), calculated using blood glucose concentration at the 0 min time point as a baseline, were calculated.

### Liver lipids and cholesterol

Liver lipids were extracted using the method of Fölch [19], solvent evaporated under nitrogen, and the extracted lipids determined gravimetrically. Liver cholesterol was quantified enzymatically at six weeks as described by Gallaher et al. [20].

### Urinary glucose, ketones and thiobarbituric acid reactive substances

Urinary glucose was determined using a modification of a previously described method [21]. Urinary β-hydroxybutyrate was quantified using an enzymatic kit (Cayman Chemical, Ann Arbor, MI). Thiobarbituric acid reactive substances (TBARS) were measured in the urine as previously described [22].

### Muscle acylcarnitines

Forty mg of frozen gastrocnemius muscle was homogenized in 1 mL deionized water. Fifty μL of the homogenate, 50 μL of 5 μmol/L heptadecanoylcarnitine internal standard (synthesized as previously described [23]) and 1 mL 80% methanol were added to a microcentrifuge tube, vortexed and centrifuged for 5 min at 13,000 *g*. Supernatants were evaporated and reconstituted in 500 μL 80% methanol. Acylcarnitines were quantitated using a LC-MS system consisting of a Shimadzu LC 10AD pump and autoinjector with a flow rate of 1 mL/min. The mobile phase was a linear gradient from 100% water to 100% methanol over 25 min followed by a hold for 10 min. The analytical column was a 100 × 4.6 mm Hypersil GOLD C18 (3 μm particle size) (Thermo Scientific, Waltham, MA) and detection was by a Quattro Micro electrospray tandem mass spectrometry (Waters Micromass, Milford, MA).

### Relative Quantitative Real-Time RT-PCR

Total RNA was extracted from tissue using TRIzol (Invitrogen, Carlsbad, CA) according to the manufacturer’s instructions and reverse transcribed to cDNA using SuperScript II Reverse Transcriptase (Invitrogen). RT-PCR was performed using SYBR Green qPCR SuperMix Universal kit (Invitrogen) with an ABI 7500 Real Time PCR System (Applied Biosciences, Foster City, CA). Each gene expression was normalized to β-actin and the average of triplicate measures were normalized to the lean control group.

### Statistical analysis

Group means were compared by one-way analysis of variance and correlation coefficients determined using SAS System 9.3 for Windows (SAS Institute, Cary, NC). Differences among groups were inspected using Duncan’s multiple range test. Viscosity measurements of intestinal contents were log transformed as they were not normally distributed. P<0.05 was taken for statistical significance. The amount of diet consumed during the meal tolerance test was used as a covariate for analysis of the tAUC to correct for small differences in diet consumption. Average daily food intake was used as a covariate for analysis of all relevant endpoints to correct for differences in food intake.

## Results

### Small intestinal contents viscosity increases with consumption of viscous fibers and decreases the postprandial glucose curve after a meal

**Figure 1:**
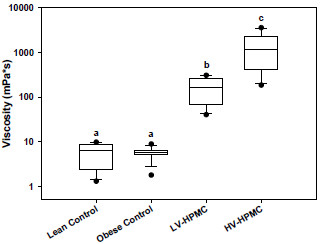
**Small intestinal contents supernatant viscosity in animals fed either cellulose or HPMC.** Values are shown as box plots, with n=10-12 for each group. The horizontal line within each box represents the median and the boundary of the boxes indicates the 25th and 75th percentile of the data. Error bars indicate 10th and 90th percentiles. Circles above or below the error bars indicate outlying data points. Values were log transformed prior to statistical analysis by one-way analysis of variance. Values not sharing a common letter are significantly different (p<0.05).

The intestinal contents supernatant viscosity of the two groups fed HPMC was much greater than the lean and obese control groups, which were fed cellulose as a fiber source (Figure 1). The tAUC of the postprandial glucose curve after a meal (Table 1) in the LV-HPMC and HV-HPMC groups was significantly less than the obese control, and the tAUC of the HV-HPMC did not differ significantly from the lean control group.

**Table 1:** **Measures of food intake, glucose control and plasma adipokines**^**1**^

**Parameter**	**Lean Control**	**Obese Control**	**LV-HPMC**	**HV-HPMC**
Mean food intake (g/day)	16.7 ± 0.2^a^	30.0 ± 1.0^d^	24.4 ± 0.6^c^	22.6 ± 0.4^b^
Meal tolerance test total tAUC (x10^3^)^2^	26.9 ± 1.3 ^a^	44.7 ± 3.9^c^	36.9 ± 2.5^b^	33.3 ± 1.8^ab^
Fasting plasma glucose (mmol/L)^3^	7.2 ± 0.2^a^	27.4 ± 1.5^c^	24.6 ± 2.5^c^	13.7 ± 1.5^b^
% Glycated Hb^4^	6.8 ± 0.3^a^	17.0 ± 1.0^c^	13.5 ± 1.1^b^	8.2 ± 0.4^a^
Urinary glucose (mg/24h)^3^	39.8 ± 5.3^a^	915.5 ± 152.6^c^	517.1 ± 121.7^b^	207.6 ± 85.6^a^
Urinary β-hydroxybutyrate (μg/24h)^3^	48.4 ± 8.3^a^	353.0 ± 75.8^c^	201.4 ± 37.2^b^	145.5 ± 26.8^ab^
Kidney weight, as percentage of body weight (g/100g BW)^4^	0.67 ± 0.01^b^	0.79 ± 0.02^c^	0.67 ± 0.02^b^	0.56 ± 0.01^a^
Glucose tolerance test, total AUC (x10^3^)^3^	22.3 ± 0.8^a^	74.8 ± 3.0^d^	56.6 ± 5.8^c^	33.8 ± 2.3^b^
Glucose tolerance test, incremental AUC (x10^3^)^3^	6.0 ± 0.7	6.8 ± 2.4	3.4 ± 2.3	3.9 ± 2.5
QUICKI^3^	0.288 ± 0.003^c^	0.206 ± 0.002^a^	0.199 ± 0.002^a^	0.218 ± 0.004^b^
Non-esterified fatty acids in fasted state (mmol/L)^3^	0.86 ± 0.05^a^	1.58 ± 0.11^b^	1.74 ± 0.19^b^	1.63 ± 0.12^b^
Non-esterified fatty acids in fed state (mmol/L)^4^	0.51 ± 0.04^a^	0.87 ± 0.04^b^	0.96 ± 0.08^b^	0.94 ± 0.06^b^
Urinary TBARS (ug/24h)^3,5^	3.9 ± 0.5^a^	7.6 ± 0.8^b^	5.2 ± 0.3^a^	4.5 ± 0.3^a^
Leptin in fasted state (ng/mL)^3^	2.2 ± 0.1^a^	50.8 ± 2.9^b^	66.1 ± 8.0^c^	52.9 ± 5.0^bc^
Adiponectin in fasted state (μg/mL)^3^	36.5 ± 4.2^b^	20.3 ± 2.4^a^	23.3 ± 3.1^a^	56.3 ± 4.3^c^
Corticosterone in fed state (ng/mL)^4^	62.7 ± 21.3	121.5 ± 26.2	75.6 ± 19.7	114.6 ± 32.9

^1^Values represent mean ± SEM, n=10-12, except where noted. Values within a row that do not share a common superscript are significantly different, p<0.05.

^2^Measured after one week of feeding of the diets.

^3^Measured after five weeks of feeding of the diets.

^4^Measured at the end of the feeding trial (6 weeks).

^5^n=8.

### Body weight increases with consumption of viscous fibers

**Figure 2:**
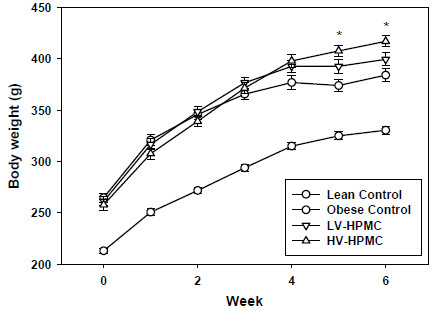
**Weekly body weight in animals fed either cellulose or HPMC.** Values represent means ± SEM, n=12. *, significantly different from obese control, p<0.05.

**Figure 3:**
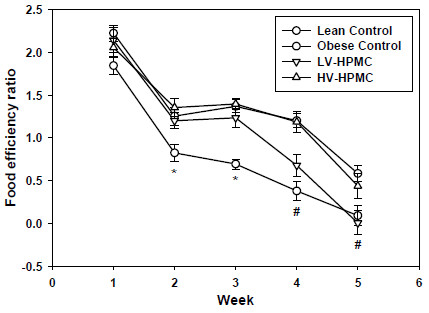
**Weekly food efficiency ratio (weight gained during week / 24 h food intake during week) in animals fed either cellulose or HPMC.** Values represent means ± SEM, n=12. Values not sharing a common letter are significantly different (p<0.05). *, obese control significantly different from lean control, LV-HPMC and HV-HPMC groups. #, obese control significantly different from lean control and HV-HPMC groups.

Rats consuming the HV-HPMC diet were significantly heavier than the obese control rats after six weeks on the experimental diets. The body weights of the three ZDF groups remained the same up to four weeks of consuming the diets but during the final two weeks the obese control group no longer gained weight (Figure 2). The obese control rats had significantly greater food intake (Table 1) than the two HPMC groups despite a lower bodyweight, although only the body weight of the HV-HPMC group was significantly greater than the obese control group. Moreover, the food efficiency ratio (weight gained during week / 24h food intake during week) was significantly greater in the HV-HPMC group compared to the obese control group for weeks 2–5 (not measured in the final week due to glucose and insulin tolerance testing) and was not different from the lean control group at any week (Figure 3).

### Consumption of viscous fibers improves markers of glucose control and insulin resistance

To investigate whether consumption of viscous fibers reduces insulin resistance in ZDF rats, markers of glucose control, NEFA and plasma adipokines were measured (Table 1). Fasting plasma glucose after five weeks was significantly lower in the HV-HPMC group compared to the obese control and LV-HPMC groups. The percentage of glycated hemoglobin, a measure of long-term glucose control, was lower in the two HPMC groups compared to the obese control, and the HV-HPMC group did not differ from the lean control. Urinary excretion of glucose and the ketone body β-hydroxybutyrate was significantly less in the two HPMC groups compared to the obese control, with a greater reduction in the HV-HPMC group, indicating that the obese control group excreted more energy through the urine (Table 1), consistent with this group’s lack of weight gain in spite of a greater food intake. Kidney weight as a percentage of body weight (relative kidney weight) is an indirect marker of blood glucose control, as it increases proportionally with increases in glycated hemoglobin [24]. Consistent with other glucose control measures, relative kidney weight was significantly less in the LV-HPMC and HV-HPMC groups relative to the obese control. Further, relative kidney weight in the HV-HPMC group was significantly less than in the lean control group. The LV-HPMC and HV-HMPC groups had a lower tAUC during the oral glucose tolerance test at five weeks on the diet compared to the obese control group, and the HV-HPMC group was lower than the LV-HPMC group. Thus insulin resistance was inversely proportional to intestinal contents viscosity. However, the glucose iAUC, which does not incorporate differences in fasting glucose concentrations, did not differ among the groups. Considering that all other measures of glucose control point to an improvement with HPMC, this suggests that calculating the total, rather than incremental AUC, may be more indicative of the degree of insulin resistance in the ZDF model of type 2 diabetes. Additionally, the surrogate index QUICKI was calculated as an estimate of insulin resistance and was found to be greater after five weeks on the diet in the HV-HPMC group compared to the obese control, indicating less insulin resistance. To determine if insulin resistance is associated with increased circulating NEFA, we measured plasma NEFA in both the fasted and fed states. Plasma NEFA concentrations were greater in all ZDF groups compared to the lean control group, but no differences were found among the ZDF groups in either state. Finally, as a marker of oxidative stress, which has also been implicated as a cause of insulin resistance [25], 24-hour urinary TBARS were measured. The two HPMC groups had a significantly lower 24-hour urinary TBARS excretion compared to the obese control, and did not differ from the lean control.

Since it has become increasingly clear that the altered endocrine activity of adipose tissue is associated with insulin resistance [26], we measured the plasma concentration of leptin and adiponectin, two central regulators of insulin resistance that are involved in maintaining energy homeostasis. The fasting plasma leptin concentrations, although dramatically elevated in all three ZDF groups, were not different between the HV-HPMC group and the obese control and surprisingly, was significantly greater in the LV-HPMC than the obese control group (Table 1). In contrast to leptin, adiponectin has a strong negative correlation with fat mass, decreasing in concentration during obesity and insulin resistance and increasing in concentration during weight loss [27]. The HV-HPMC group displayed a significantly greater concentration of fasting plasma adiponectin than the obese control group. However, the LV-HPMC group did not differ from the obese control group. Overall, plasma adiponectin in the fed state showed a significant correlation with insulin resistance, measured as QUICKI (r=0.62, p<0.001). Interestingly, plasma adiponectin in the fasted state did not correlate with insulin resistance (r=0.26, p=0.09). Elevated plasma concentrations of glucocorticoids have been associated with many of the components of metabolic disease, including hyperglycemia, insulin resistance, central obesity and fatty liver [28]. Studies of plasma corticosterone concentrations in obese ZDF rats, compared to lean controls, have been inconsistent, with both an elevation [29] or no difference [30] reported. In the present study, only a slight but statistically non-significant increase was found in the obese control group compared to the lean control group, and neither HPMC group differed from the obese control group.

### Viscous fibers decrease visceral fat pad weight

**Figure 4:**
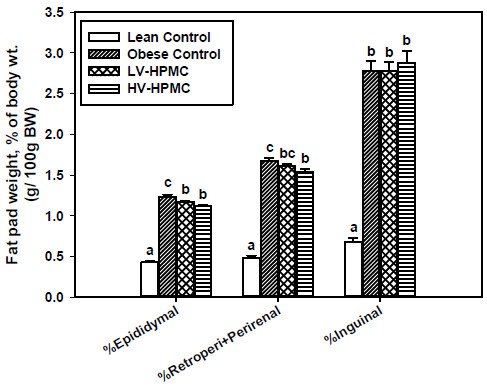
**The ratio of fat pad weight to total body weight after consumption of experimental diet for 6 weeks in animals fed either cellulose or HPMC.** Values represent means ± SEM, n=12. Values not sharing a common letter are significantly different (p<0.05).

As a percentage of final body weight, the obese control group had greater visceral fat pad weights than the lean control group, as expected (Figure 4). However, the group fed HV-HPMC had lighter epididymal and retroperitoneal+perirenal fat pad weights as a percentage of final body weight, compared to the obese control (Figure 4). These fat pads represent visceral fat. In contrast, there were no significant differences among the ZDF groups in inguinal fat pad weight as a percentage of final body weight, which represents subcutaneous fat.

Since food intake varied among the groups, the potential influence of food intake on measures of glucose control and adiposity was examined statistically. Food intake was used as a covariate in the analysis of variance model to indicate whether food intake was a significant explanatory factor. Average food intake was not a statistically significant covariate for any relevant measure of glucose control or fat pad weight as a percentage of body weight except 24-hour urinary glucose excretion. Rather than a direct relationship between food intake and urinary glucose, it is likely that food intake is an indirect measure of insulin resistance and that insulin resistance is more directly associated with urinary glucose. Indeed there is a highly significant correlation between urinary glucose and the glucose tolerance test area under the curve (r=0.623, p=0.0002), a measure of insulin resistance not directly influenced by food intake. Based on this statistical analysis, average food intake does not appear to explain the differences in insulin resistance or adiposity among the ZDF groups.

### Viscous fibers reduce fatty liver

The average liver weight of the LV-HPMC group was significantly less than the obese control and liver weight in the HV-HPMC group was significantly less than the LV-HPMC group (Table 2). To investigate whether the increased liver weight in the obese control group was due to the development of fatty liver, total liver lipids were measured. Total liver lipid was greatly increased in the obese control group compared to the lean control group. There was no difference in total liver lipid concentration between the LV-HPMC group and obese control but total liver lipid was reduced in the HV-HPMC group compared to the obese control and LV-HPMC groups. Among the ZDF groups, total liver lipid was strongly but inversely correlated with insulin resistance, as measured by the QUICKI index (r = −0.71, p<0.001). Liver cholesterol concentration was similar among the lean control, obese control and LV-HPMC groups, but was significantly lower in the HV-HPMC group compared to the obese control group. Although fasting plasma triacylglycerols were greatly elevated in all ZDF groups compared to the lean control group, there were no significant differences among the ZDF groups; however, there was a trend for reduced triacylglycerols in the HV-HPMC group (p=0.10) compared to the obese control group (Table 2).

**Table 2:** **Liver weights, liver lipids, liver cholesterol and plasma TAG**^**1**^

**Parameter**	**Lean Control**	**Obese Control**	**LV-HPMC**	**HV-HPMC**
Liver weight (g)	9.3 ± 0.2^a^	22.6 ± 0.7^d^	20.7 ± 0.7^c^	16.4 ± 0.7^b^
Liver lipid (g)	0.40 ± 0.02^a^	3.53 ± 0.46^c^	3.16 ± 0.25^c^	1.81 ± 0.24^b^
Liver cholesterol (mg/g)	4.7 ± 0.2^ab^	5.0 ± 0.4^b^	4.9 ± 0.2^ab^	4.0 ± 0.4^a^
Fasting plasma TAG (mmol/L)^2^	0.6 ± 0.1^a^	5.9 ± 0.7^b^	5.6 ± 1.2^b^	4.1 ± 0.7^b^

^1^Values represent mean ± SEM, n=12. Values within a row that do not share a common superscript are significantly different, p<0.05.

^2^Measured after five weeks of feeding of the diets.

Fat accumulation in the liver is a common finding in obesity and type 2 diabetes and is strongly associated with insulin resistance [2]. Hepatic insulin resistance in turn is associated with increased rates of gluconeogenesis and lipogenesis. In the present study, there was a trend towards a reduction in gene expression of the gluconeogenic enzymes phosphoenolpyruvate carboxykinase (PEPCK) and glucose-6-phosphatase (G6Pase) in the HV-HPMC group compared to the obese control (Table 3), consistent with the improvement in insulin resistance in this group. Next, to elucidate the possible mechanism by which HPMC reduced fatty liver, we measured the gene expression of enzymes and transcription factors involved in hepatic lipogenesis. Gene expression of fatty acid synthase (FAS) was greater in the obese control group compared to the lean control group, but the HV-HPMC group did not differ from the obese control group. Expression of sterol regulatory element binding protein 1c (SREBP-1c), a master regulator of lipogenesis [31], did not differ among the groups. Finally, expression of carnitine palmitoyl transferase 1α (CPT-1α), the rate-limiting step in hepatic fatty acid oxidation, was significantly increased in the obese control compared to the lean control. There was a strong trend for greater expression of CPT-1α in the HV-HPMC group compared to the obese control (p=0.061).

**Table 3:** **Expression of genes in liver and gastrocnemius muscle**^**1**^

**Gene**^**2**^	**Lean Control**	**Obese Control**	**HV-HPMC**
mRNA/Actin	Liver		
PEPCK	1.4 ± 0.3	1.0 ± 0.3	0.7 ± 0.1^#^
G6Pase	1.2 ± 0.2^a^	2.8 ± 0.4^b^	2.0 ± 0.4^ab^
SREBP-1c	1.1 ± 0.3	1.5 ± 0.4	1.2 ± 0.2
FAS	1.3 ± 0.3^a^	11.9 ± 1.4^b^	10.4 ± 0.9^b^
CPT-1α	1.3 ± 0.2^a^	2.0 ± 0.6^b^	2.7 ± 0.3^b*^
	Gastrocnemius Muscle		
CPT-1β	1.3 ± 0.2^a^	3.3 ± 0.6^b^	3.3 ± 0.8^b^
PGC-1α	1.1 ± 0.2^a^	2.1 ± 0.5^b^	1.5 ± 0.2^ab^
UCP3	1.0 ± 0.2	1.3 ± 0.4	0.8 ± 0.2

^1^ Values represent mean ± SEM, n=5-6 for SREBP-1c and 10–12 for all other values. Values within a row that do not share a common superscript are significantly different, p<0.05. #, p=0.062 compared to lean control. *, p=0.061 compared to the obese control.

^2^ Abbreviations are: *PEPCK*, phosphoenolpyruvate carboxykinase; *G6Pase*, glucose-6-phosphatase; *SREBP-1c*, sterol regulatory element-binding protein 1c; *CPT-1α*, carnitine palmitoyltransferase 1α; *FAS*, fatty acid synthase; *CPT-1β*, carnitine palmitoyltransferase 1β; *PGC-1α*, peroxisome proliferator-activated receptor coactivator 1α; *UCP3*, uncoupling protein 3;.

### Decreased insulin resistance by dietary intervention is not associated with decreased concentration of acylcarnitines

**Figure 5:**
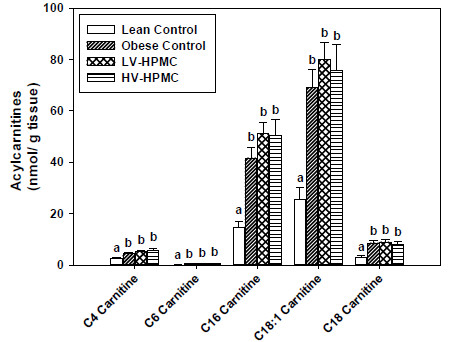
**Acylcarnitine concentrations in gastrocnemius muscle of animals fed either cellulose or HPMC.** Values represent means ± SEM, n=10-12. Values not sharing a common letter are significantly different (p<0.05).

Acylcarnitines accumulate in the muscle when fatty acids imported into the mitochondria via carnitine palmitoyltransferase 1β (CPT-1β) exceed the capacity of β-oxidation. Acylcarnitine concentration in the muscle has been shown to increase in ZDF rats compared to lean rats and may be a marker for increased fatty acid β-oxidation [32]. In the present study, the concentration of acylcarnitines in the muscle was increased in all three ZDF groups compared to the lean control but the LV-HPMC and HV-HPMC groups did not differ from the obese control (Figure 5). To investigate further the relationship between acylcarnitine concentration and fatty acid oxidation we examined gene expression of CPT-1β, peroxisome proliferator-activated receptor gamma coactivator 1-α (PGC-1α) and uncoupling protein 3 (UCP3) in the muscle. The obese control and HV-HPMC groups had greater expression of CPT-1β compared to the lean control; however there was no difference between the HV-HPMC and obese control groups (Table 3). PGC-1α expression was also significantly greater in the obese control group compared to the lean control, with expression in the HV-HPMC intermediate between these two groups. There were no significant differences in UCP3 expression among the groups.

## Discussion

Our results indicate that chronic consumption of the viscous, non-fermentable fiber HPMC can decrease diabetic wasting, improve insulin resistance and reduce the development of fatty liver in a model of obesity with type 2 diabetes. The HPMC-containing diets delayed the absorption of glucose by the intestine, as indicated by the decreased postprandial glucose curve after a meal. Since it has been shown that viscous fibers decrease the postprandial glucose curve and possibly ameliorate insulin resistance in normal mice and hamsters [33,34], we assessed their effect in a model of obesity with diabetes, the ZDF rat.

The consumption of HPMC by ZDF rats slowed the progression of the diabetic phenotype as evidenced by improved glucose control and decreased insulin resistance as well as reducing other associated conditions such as oxidative stress and glycosuria. The decreased plasma glucose tAUC during the oral glucose tolerance test and percent glycated hemoglobin in both HPMC groups, coupled with an increased QUICKI index in the HV-HPMC group, indicate greater glucose control and less insulin resistance and demonstrate that a high intestinal contents viscosity limits the progression of insulin resistance in this animal model. Moreover, there was a significant viscosity-dependent decrease in relative kidney weight during consumption of the HPMC diets when compared to the obese control. During the initial stage of diabetes, renal hypertrophy occurs proportional to glycemic control [35], which is considered an early stage in the development of diabetic nephropathy. The only marker not to indicate a statistically significant improvement in glucose control with viscous fiber was the iAUC. However, the values were highly variable, due in part to large differences in fasting plasma glucose. Thus, the iAUC may not capture differences in insulin resistance as well as other measures. In this model of advanced type 2 diabetes, it appears that the tAUC may be a better predictor of insulin resistance. Thus, in the ZDF rat, consumption of a viscous fiber greatly improves glycemic control and reduces insulin resistance, and appears to do so in proportion to intestinal contents viscosity.

Paradoxically, the HV-HPMC group had a greater final body weight but lower food intake than the obese control group. Others have reported treatments in ZDF rats that decreased food intake but led to either increased [36] or no change in body weight [37]. As indicated by the food efficiency ratio of the four groups, the HV-HPMC group was able to more efficiently use the energy consumed compared to the obese control group. The food efficiency ratio was not different during the first week but the obese control was significantly lower than the HV-HPMC groups in weeks 2–5. This could be due to either increased energy expenditure, decreased intestinal absorption of macronutrients, or increased excretion of energy in the obese control group. It is not apparent how HPMC treatment would decrease energy expenditure or increase absorption of macronutrients relative to the obese control group. Therefore it is more likely that the obese control group lost more energy in the urine. This may be explained by the progression of insulin resistance resulting in increased excretion of glucose and ketone bodies in the urine in the obese control group, a known result of untreated type 2 diabetes. The difference in food efficiency ratio did not result in a body weight difference in weeks 2 and 3 but the obese control and HV-HPMC groups gradually separated and were significantly different in the last two weeks. This suggests that the HV-HPMC had decreased diabetic wasting and experienced normalized body growth while the obese control group could not maintain a normal growth curve. As expected, the increased small intestinal viscosity from HPMC was inversely related to the 24-hour urinary excretion of glucose (r=−0.54, p=0.001, as logarithm of viscosity vs. glucose excretion) and β-hydroxybutyrate (r=−0.41, p=0.02, as logarithm of viscosity vs. β-hydroxybutyrate excretion) in the ZDF groups. However, given the magnitude of the difference in food intake, other factors, such as differences in physical activity, may be involved.

A decreased postprandial glucose response will reduce the plasma insulin response, which may lead to reduced tissue lipid accumulation by decreasing lipogenesis or increasing fatty acid β-oxidation. Differences in visceral fat pad weight, while statistically significant, were small and would have contributed little to differences in body weight. However, the HV-HPMC group, which was significantly heavier than the obese control, had the lightest visceral fat pad weight as a percent of final body weight, indicating a change in body composition. Similarly, Syrian hamsters on a high fat diet supplemented with HPMC also had reduced abdominal fat with no change in body weight, further supporting an effect of HPMC on reducing adiposity [34].

The circulating concentration of NEFA has been postulated to play a role in muscle insulin resistance, possibly through oxidative stress and mitochondrial dysfunction [38]. Neither fasting nor fed NEFA levels differed among the three ZDF groups despite large differences in insulin resistance, suggesting that the increased insulin sensitivity may not be directly related to plasma NEFA in this model, but rather by other factors such as circulating adipokines. One adipokine, leptin, is typically positively correlated with fat mass [39], however in the ZDF model of extreme insulin resistance and a defective leptin receptor, this correlation is lost [40], a finding confirmed in the present experiment (r=0.0703, p=0.65). Adiponectin is another circulating adipokine that correlates well with whole-body insulin sensitivity [38] and is decreased in subjects with type 2 diabetes [41,42]. In a cross-sectional study, cereal fiber intake associated with higher levels of plasma adiponectin in diabetic men [43] and Zucker rats consuming soluble cocoa fiber had higher levels of adiponectin compared to rats on a diet containing only cellulose [44]. In this study, the HV-HPMC group had the highest concentration of adiponectin and greatest insulin sensitivity. Adiponectin is thought to inhibit hepatic gluconeogenesis and increase fatty acid oxidation in the muscle through increased AMP kinase and PPARα activity [45], yet we saw only a tendency for a difference in hepatic expression of G6Pase, a trend for a decrease in PEPCK and no difference in CPT-1β expression or acylcarnitine concentration in the muscle.

Livers from the obese control group contained considerably more lipid than those of the lean control group, indicating hepatic steatosis, as reported by others in this animal model [46]. Hepatic steatosis is a result of an imbalance in fatty acid uptake or synthesis versus fatty acid oxidation or export via VLDL. It is considered the first step towards development of nonalcoholic fatty liver disease. This ectopic accumulation of lipid has been strongly linked to increased insulin resistance [47-49], as was found in the obese control group in the present study. The HV-HPMC group had significantly less total liver lipids compared to the obese control group, as well as reduced insulin resistance, measured by both the QUICKI index and the glucose tolerance test, suggesting that the reduction in insulin resistance in this group led to a reduction in hepatic lipid accumulation. Low plasma adiponectin concentrations have been linked to insulin resistance [50,51], and plasma adiponectin concentrations are inversely related to hepatic steatosis [52-54], although some evidence indicates that the effect of adiponectin on hepatic steatosis is independent of insulin resistance [52,53]. The HV-HPMC group, which had the lowest insulin resistance and least hepatic steatosis, also displayed the highest plasma adiponectin concentrations. The antisteatotic effect of adiponectin, mediated through the AdipoR1 and R2 receptors [55], appears to be due to activation of AMP kinase, leading to increased fatty acid oxidation [56]. Increases in hepatic CPT1α, the rate-limiting enzyme in β-oxidation [57], resulting in increased fatty acid oxidation, have been shown to reduce liver TAG in both lean and obese rats [58]. Activation of AMP kinase also decreases expression of hepatic gluconeogenic enzymes such as PEPCK and G6Pase [59]. This is consistent with findings from the present study, in which the HV-HPMC group, with the highest plasma adiponectin, had the lowest hepatic lipid concentration, the highest hepatic expression of CPT1α, and a trend towards a reduction in the gluconeogenic enzymes PEPCK and G6Pase. However, no differences were found in the expression of FAS or of SREBP-1c, a transcription factor regulating expression of lipogenic genes, in the HV-HPMC group compared to the obese control. Others have reported decreased hepatic gene expression of FAS and SREBP-1c in Syrian hamsters fed HPMC [60]. However, these animals were not insulin resistant. Given the lack of differences between the obese control group and the HV-HPMC group in plasma fatty acids (in either the fasted or fed state), in plasma TAG, or in markers of hepatic lipogenesis, coupled with greater expression of CPT1α in the HV-HMPC group, it seems most likely that an increase in hepatic fatty acid oxidation in the HV-HPMC group is responsible for the observed decrease in hepatic lipid concentration in this group.

One current theory of the progression of skeletal muscle insulin resistance in diabetes is that accumulation of intramuscular lipids will disrupt insulin signaling pathways and decrease glucose uptake [61]. It is now believed that it is not the accumulation of triacylglycerols in the muscle tissue that is the cause of insulin resistance, but rather the generation of lipid metabolites such as ceramides, diacylglycerols and acylcarnitines that produces insulin resistance [62]. In the first and rate-limiting step of β-oxidation, fatty acyl-CoAs are attached to carnitine by the enzyme CPT-1β, allowing transport through the mitochondrial membrane [57]. However, if the energy state in the cell is high, enzymes in the electron transport chain may not increase activity sufficiently to compensate for the increased influx of acylcarnitines via CPT-1β [63]. As a result, the concentration of intracellular acylcarnitines increases. This elevated concentration has been proposed as a possible link to insulin resistance [32]. Indeed, higher levels of plasma acylcarnitines, resulting from the intracellular accumulation of acylcarnitines, are associated with insulin resistance in both humans and rodents [32,64]. Our results show increased short and long chain acylcarnitines in the three ZDF groups compared to the lean control but, surprisingly, the HV-HPMC group, which displayed less insulin resistance compared to the obese control group, as shown by an improved OGTT and a higher QUICKI, did not differ from the obese control in acylcarnitine concentration in the muscle. A previous study associating increased acylcarnitines with insulin resistance compared models displaying very large differences in insulin resistance and obesity [32]. Although acylcarnitine levels appear to increase during insulin resistance and obesity, it may be that they are only elevated as a function of other characteristics of the model, such as increased fatty acid β-oxidation, and may not be directly related to insulin resistance. Although the concentration of muscle acylcarnitines did not differ between the HV-HPMC and obese control despite differences in insulin resistance, it is conceivable that differences in the rate of β-oxidation may exist. To that end, we measured gene expression in muscle of CPT-1β and UCP3, two genes regulating fatty acid oxidation, but found no change with HV-HPMC consumption. However PGC-1α, a transcriptional coactivator linked to lipid oxidation, did trend lower, implying a decrease in fatty acid oxidation. Therefore, these results show that even though muscle acylcarnitines increased in a situation of greatly increased insulin resistance, as seen when comparing the lean and obese control groups, it appears that moderate decreases in insulin resistance, as produced by the HPMC-containing diets, were insufficient to decrease acylcarnitine concentrations.

## Conclusions

In summary, we report that chronic consumption of the viscous but non-fermentable fiber HPMC ameliorates many characteristics of diabetes, providing a reduction in insulin resistance and in wasting, as well as reducing visceral adiposity and increasing the plasma concentration of the adipokine adiponectin. This was accompanied by a decrease in liver lipids and a trend toward a decrease in markers of hepatic gluconeogenesis and an increase in a marker of fatty acid oxidation. We also report that muscle acylcarnitine concentrations remain unchanged, despite large differences in insulin resistance, suggesting that they may not correlate with insulin resistance during less extreme modifications of obesity and diabetes. These results indicate that modifying intestinal contents viscosity by the chronic consumption of viscous fibers will not only improve insulin resistance but decrease adiposity and fatty liver.

## Abbreviations

HPMC: Hydroxypropyl methylcellulose; SCFA: Short chain fatty acid; HV-HPMC: High viscosity HPMC; iAUC: Incremental area under the curve; LV-HPMC: Low viscosity HPMC; OGTT: Oral glucose tolerance test; QUICKI: Quantitative insulin sensitivity check index; tAUC: Total area under the curve; ZDF: Zucker Diabetic Fatty.

## Competing interests

D. A. Brockman, X. Chen and D. D. Gallaher have no conflicts of interest.

## Authors’ contributions

DAB, XC, and DDG designed the research; DAB conducted the research; DAB and DDG analyzed the data; DAB wrote the paper; and DDG had final responsibility for the content. All authors have read and approved the final manuscript.
